# Importance sampling method of correction for multiple testing in affected sib-pair linkage analysis

**DOI:** 10.1186/1471-2156-4-S1-S73

**Published:** 2003-12-31

**Authors:** Alison P Klein, Ilija Kovac, Alexa JM Sorant, Agnes Baffoe-Bonnie, Betty Q Doan, Grace Ibay, Erica Lockwood, Diptasri Mandal, Lekshmi Santhosh, Karen Weissbecker, Jessica Woo, April Zambelli-Weiner, Jie Zhang, Daniel Q Naiman, James Malley, Joan E Bailey-Wilson

**Affiliations:** 1 Inherited Disease Research Branch, NHGRI, NIH, Baltimore, Maryland, USA; 2 Fox Chase Cancer Center, Philadelphia, Pennsylvania, USA; 3 CIDR, Johns Hopkins Medical School, Baltimore, Maryland, USA; 4 Department of Genetics, Louisiana State University Health Sciences Center, New Orleans, Louisiana, USA; 5 Department of Psychiatry and Neurology and the Hayward Genetics Program, Tulane University, New Orleans, Louisiana, USA; 6 Department of Epidemiology, Bloomberg School of Public Health, Johns Hopkins University, Baltimore, Maryland, USA; 7 Department of Mathematical Sciences, Johns Hopkins University, Baltimore, Maryland, USA; 8 Center for Information Technology, NIH, Bethesda, Maryland, USA

## Abstract

Using the Genetic Analysis Workshop 13 simulated data set, we compared the technique of importance sampling to several other methods designed to adjust p-values for multiple testing: the Bonferroni correction, the method proposed by Feingold et al., and naïve Monte Carlo simulation. We performed affected sib-pair linkage analysis for each of the 100 replicates for each of five binary traits and adjusted the derived p-values using each of the correction methods. The type I error rates for each correction method and the ability of each of the methods to detect loci known to influence trait values were compared. All of the methods considered were conservative with respect to type I error, especially the Bonferroni method. The ability of these methods to detect trait loci was also low. However, this may be partially due to a limitation inherent in our binary trait definitions.

## Background

When many tests are conducted in a genome scan, with or without fine mapping, it is important to correct the observed *p*-values to ensure that the empirical rate of false-positive tests is equal to the desired significance level. Traditional approaches to this problem are 1) the Bonferroni method [1], which is known to be conservative, particularly when individual tests are correlated; 2) adjusting for the prior probability of linkage [2]; and 3) assuming an infinitely dense map of markers [3]. Several authors have suggested that assuming an infinitely dense map of markers is also too conservative [4,5] and that it is more appropriate to adjust for the actual number of tests performed and for the nonindependence of tightly linked markers. Using the Genetic Analysis Workshop 13 (GAW13) simulated data set, we applied the statistical technique of importance sampling (IS) to affected sib-pair (ASP) linkage tests in a genome-wide scan [6]. The IS algorithm provides an efficient tool for approximating *p*-values using various assumptions about the arrangements of markers. A key feature of this algorithm is that the marker arrangement can be completely user-specified. In particular, it is not necessary to assume that the markers are equally spaced, which is important because the *p*-value is sensitive to the degree of marker clustering.

We compared the IS approach to the Bonferroni correction and to the method proposed by Feingold et al. [7], which is based on the theory of large deviations in the context of stochastic processes and tends to avoid the conservatism of assuming an infinitely dense marker map. The Feingold et al. approach (FBS) assumes markers are equally spaced, but the IS method does not make this assumption. We were also interested in determining the extent to which IS improves on naïve Monte Carlo (NS) sampling for problems of practical interest. Therefore, we included an NS approach (equally accurate but often less computationally efficient than IS) that generates samples of normalized ASP test statistics, under the null hypothesis, using its approximating Gaussian distribution. In addition to comparing false positive rates across these adjustment methods, we also examined the effect of applying these different corrections on our power to detect trait loci. In making these comparisons, we used our knowledge of the underlying genetic model and the locations of the trait loci.

## Methods

Because our research did not seek to examine the impact of missing data, we utilized all phenotype data in both cohorts and the complete genotype data in the 100 simulated data replicates provided. We used standard clinical criteria [8,9] to define binary traits as follows: 1) high blood pressure (hibpr): affected if diagnosed with or treated for high blood pressure at any visit; 2) high cholesterol (hichl): affected if total cholesterol ≥ 240 mg/dl at any visit; 3) low high-density lipoprotein cholesterol (lohdl): affected if HDL-C < 40 mg/dl at any visit; 4) high HDL-C (hihdl): affected if HDL-C ≥ 60 mg/dl at any visit; 5) high body mass index (hibmi): affected if body mass index (BMI = 703 × weight in pounds/square of height in inches) ≥ 30 at any visit. The number of visits was not consistent across individuals; however, individuals were coded as unknown for a trait only if they had no observations for that trait at any visit. Based on these criteria, the mean number of ASPs across all replicates for each trait was: 1) hibpr 668 (range 562–802), 2) hichl 209 (range 146–275), 3) lohdl 209 (range 158–272), 4) hihdl 301 (range 232–373), 5) hibmi 350 (range 253–451).

The genome-wide scan for each replicate consisted of 399 markers. ASP linkage analysis was performed using GENIBD and SIBPAL [10] for each trait with each marker locus. The mean proportion of alleles shared IBD () was computed from complete nuclear family information using one marker at a time (single point). The standard ASP test statistic



for *N *affected sib pairs was computed, and unadjusted *p*-values were obtained by comparing this statistic with a standard normal distribution [11]. This same statistic was also used by each of the adjustment methods to compute *p*-values corrected for the 399 tests. The two sampling approaches use the sex-averaged marker map, assuming a Haldane mapping function, to derive correlations in the linkage test results between adjacent markers, and they also assume Hardy-Weinberg equilibrium is in effect. These methods provide an estimate of the exceedance probability, the probability that one or more test statistics exceeded a particular observed value. These methods are based on averaging the results of independent random realizations of the same sampling experiment and give an unbiased estimate of a true exceedance probability whose variance is inversely proportional to the Monte Carlo sample size (in this study 100,000). IS differs from NS in that samples are drawn conditionally on at least one marker test statistic exceeding the threshold value. Because of this, IS is more computationally efficient when the target exceedance probability is small and the number of tests is large. Additionally, since the IS algorithm performs extremely well for small *p*-values, when ordinary Monte Carlo sampling tends to break down, we now have at our disposal a tool for quantifying the effect of marker clustering on the true *p*-value, and for determining the quality of a large deviation approximation given by Feingold et al. [7]. Adjusted *p*-values were also computed using the Bonferroni and FBS methods.

To examine the rate of false positives for each method, we considered all marker loci on the even-numbered chromosomes, a total of 197 markers, which are known to be unlinked to these traits. To examine the effect of these correction methods on the power to detect trait loci, we considered markers flanking known trait loci. We selected only trait loci that contributed at least 10% to baseline effects of the underlying quantitative trait.

## Results

Tables 1 and 2 show type I error results for the unadjusted ASP tests (UN) and for the various methods used to correct for multiple tests: Bonferroni (BF), FBS, NS, and IS. Replicate-wise type I error rates (Table 1) are computed as the proportion of the 197 unlinked marker tests giving a *p*-value of < 0.05, averaged over 100 replicates. Experiment-wise type I error rates (Table 2) are shown in terms of the number of replicates (out of 100) for which at least one of the 197 marker tests results in a *p*-value less than the specified significance level. Within replicates, the observed proportion of unlinked markers on the even-numbered chromosomes that showed significant linkage at the 0.05 level was less than 5% on average for the unadjusted tests (Table 1). However, the experiment-wise probability of observing at least one unlinked marker significant at the 0.05 level was close to 1 for all five traits when no adjustment was made for multiple tests (Table 2). However, all of the correction methods appeared to be conservative, especially the Bonferroni method.

The results of the power analysis for the markers nearest to selected trait loci are presented in Table 3, which shows the number of replicates (out of 100) for which each marker test gave a result significant at the specified level. The greatest observed power to detect linkage was observed for hibmi locus b11 with marker c13g7, which are only 0.56 cM apart. This trait locus was simulated to contribute 40% of the variance of the baseline value. At the 0.05 significance level, we were able to detect this locus with a marker < 1 cM away in 87% of the replicates without correcting for multiple tests and in 15–17% of the replicates when correcting for multiple tests. While moderate power to detect some of the other trait loci (on chromosome 21 for hibpr and on chromosome 9 for lohdl) was observed before adjustment for multiple tests, there was virtually no power after adjustment for multiple tests or at more stringent significance levels. When any difference in power was observed between the various correction methods, the Bonferroni correction had lower power.

Compared with naïve sampling, IS is much more efficient, in terms of the precision of the estimate (small variance) it can produce with a given number of iterations, when the exceedance probability estimated is small and the number of tests is large. In this study, correcting for the 399 tests, the relative efficiency



was at least 189 for an exceedance probability of < 0.05, corresponding to a z ≥ 3.613 (see Table 4).

## Discussion

These results show that the observed experiment-wise type I error rates are somewhat conservative when using any of the considered methods of adjustment for multiple tests, but that the FBS, NS, and IS methods are slightly less conservative than the Bonferroni method. However, the limited number of replicates available for study makes it difficult to make definitive statements about these methods.

The standard normal approximation to the standard ASP test statistic is valid under the null hypothesis when markers are fully informative. The fact that we obtain unadjusted *p*-values that are around 0.03 instead of the nominal 0.05 suggests that when estimates of sharing come from SIBPAL, which makes imputations about sharing for non-fully informative markers, the standard normal approximation is conservative (as expected).

While power was generally low in these data and may have been affected by the loss of information inherent in our binary trait definitions, we were able to show that the IS (i.e., computationally efficient NS) method of correcting for multiple tests exhibited the same or greater power than the FBS and Bonferroni methods, while still adequately controlling type I error. When there is a marked irregularity in the spacing between markers, as would be observed in a genome-wide scan followed by fine mapping in one or several regions, the IS method has been shown to perform significantly better than the FBS method [6]. The increased efficiency of the IS method as compared with the naïve Monte Carlo method facilitates its application to the analysis of genome scan and fine mapping data. However, any Monte Carlo sampling method is computationally intensive, and we recommend using it only when there is the possibility of significance indicated by an uncorrected test. The results of the IS correction, as with any Monte Carlo method, depend on the allele frequencies and the assumptions of the map function and Hardy-Weinberg Equilibrium. Additional simulations are needed to further compare these methods under varying conditions.

The critical values of the NS method, as shown in Table 4, are slightly higher than those obtained for IS. This is because the same number of iterations (100,000) was used for both methods. The IS method, being more efficient around the critical value, was more precise. We used a conservative approach in selecting a critical value of z such that all scores at least this high produced a *p*-value below the desired significance level.

## Figures and Tables

**Table 1:** Replicate-wise type I error rates: mean proportion of markers per replicate having *p*-values < 0.05.

**Trait**	**UN**	**BF**	**FBS**	**NS**	**IS**
hibpr	0.02959	0	0.00010	0.00010	0.00010
hichl	0.02802	0.00005	0.00005	0.00005	0.00005
lohdl	0.03513	0.00010	0.00015	0.00015	0.00015
hihdl	0.03066	0	0	0	0
hibmi	0.03223	0.00010	0.00010	0.00010	0.00010

**Table 2:** Experiment-wise type I errors: number of replicates with at least one significant marker.

Trait	**α = 0.05**					**α = 0.01**					**α = 0.001**				
			
	**UN**	**BF**	**FBS**	**NS**	**IS**	**UN**	**BF**	**FBS**	**NS**	**IS**	**UN**	**BF**	**FBS**	**NS**	**IS**
hibpr	99	0	2	2	2	55	0	0	0	0	8	0	0	0	0
hichl	97	1	1	1	1	51	0	0	0	0	6	0	0	0	0
lohdl	99	2	3	3	3	52	0	0	0	0	6	0	0	0	0
hihdl	97	0	0	0	0	60	0	0	0	0	9	0	0	0	0
hibmi	99	2	2	2	2	49	0	1	1	1	9	0	0	0	0

**Table 3:** Number of replicates in which the marker nearest to trait locus was significant.

**Trait Locus**	**Marker (Distance to Trait, cM)**	**α = 0.05**					**α = 0.01**					**α = 0.001**				
				
		**UN**	**BF**	**FBS**	**NS**	**IS**	**UN**	**BF**	**FBS**	**NS**	**IS**	**UN**	**BF**	**FBS**	**NS**	**IS**
**hibpr**																
b34	c5g22 (1.5)	4	0	0	0	0	0	0	0	0	0	0	0	0	0	0
	c5g23 (12.1)	3	0	0	0	0	0	0	0	0	0	0	0	0	0	0
b38	c7g1 (4.3)	4	0	0	0	0	1	0	0	0	0	0	0	0	0	0
b35	c13g8 (1.4)	6	0	0	0	0	2	0	0	0	0	0	0	0	0	0
	c13g9 (5.7)	3	0	0	0	0	0	0	0	0	0	0	0	0	0	0
b37	c21g3 (2.6)	43	0	1	1	1	23	0	0	0	0	3	0	0	0	0
	c21g4 (10.9)	50	1	1	1	1	23	0	0	0	0	5	0	0	0	0
**hichl**																
b31	c1g17 (5.8)	9	0	0	0	0	3	0	0	0	0	0	0	0	0	0
	c1g18 (8.5)	3	0	0	0	0	0	0	0	0	0	0	0	0	0	0
b30	c11g8 (3.0)	10	0	0	0	0	3	0	0	0	0	0	0	0	0	0
	c11g9 (16.5)	7	0	0	0	0	1	0	0	0	0	0	0	0	0	0
b32	c15g13 (10.8)	12	0	0	0	0	2	0	0	0	0	0	0	0	0	0
	c15g14 (9.7)	7	0	0	0	0	2	0	0	0	0	0	0	0	0	0
**lohdl**																
b12	c9g1 (5.2)	23	0	0	0	0	3	0	0	0	0	0	0	0	0	0
b20	c17g6 (3.4)	15	0	0	0	0	2	0	0	0	0	0	0	0	0	0
	c17g7 (10.4)	13	0	0	0	0	4	0	0	0	0	0	0	0	0	0
**hihdl**																
b12	c9g1 (5.2)	14	0	0	0	0	4	0	0	0	0	0	0	0	0	0
b20	c17g6 (3.4)	10	0	0	0	0	1	0	0	0	0	0	0	0	0	0
	c17g7 (10.4)	10	0	0	0	0	3	0	0	0	0	0	0	0	0	0
**hibmi**																
b1	c5g12 (6.3)	6	0	0	0	0	1	0	0	0	0	1	0	0	0	0
	c5g13 (11.9)	5	0	0	0	0	3	0	0	0	0	0	0	0	0	0
b2	c7g20 (7.2)	3	0	0	0	0	1	0	0	0	0	0	0	0	0	0
	c7g21 (5.1)	3	0	0	0	0	0	0	0	0	0	0	0	0	0	0
b11	c13g7 (0.6)	87	15	17	17	17	55	7	7	7	7	31	1	3	1	3
	c13g8 (13.2)	46	1	1	1	1	18	0	0	0	0	3	0	0	0	0

**Table 4:** Critical values of z statistic for each method and significance level.

	α = 0.05	α = 0.01	α = 0.001
Uncorrected	1.645	2.327	3.091
Bonferroni	3.662	4.056	4.565
FBS	3.627	4.028	4.536
NS	3.614	4.027	4.572
IS	3.613	4.024	4.545
